# Comparative Analysis of Functional Adaptations to Vivifrail Intervention Between Higher- and Lower-Fitness Healthy Older Adults

**DOI:** 10.3390/life15070988

**Published:** 2025-06-20

**Authors:** Fang-Ru Lee, Yu-Jui Li, Chia-Yu Tang, Chang-Chi Lai, Hsia-Ling Tai, Szu-Kai Fu

**Affiliations:** 1 Graduate Institute of Sports Training, College of Kinesiology, University of Taipei, Taipei City 111036, Taiwan; tcc9913@tcc.gov.tw (F.-R.L.); jimmy_lin19@yahoo.com.tw (C.-Y.T.); danatai1008@gmail.com (H.-L.T.); 2 Department of Recreation and Sports Management, College of Kinesiology, University of Taipei, Taipei City 111036, Taiwan; li542058@utaipei.edu.tw; 3 Department of Exercise and Health Sciences, College of Kinesiology, University of Taipei, Taipei City 111036, Taiwan; sportsinjury0406@gmail.com; 4 Department of Physical Education, College of Science, University of Taipei, Taipei City 100234, Taiwan

**Keywords:** Vivifrail, functional fitness, older women, muscle strength, arterial stiffness, exercise intervention, dual-task training

## Abstract

This study evaluated the differential effects of an advanced Vivifrail D-level exercise intervention on arterial stiffness, functional fitness, and body composition in robust older women with varying baseline fitness levels. A total of 41 participants were assigned to a higher-fitness group (HFG, *n* = 22) or a lower-fitness group (LFG, *n* = 19) based on their 30 s chair stand performance. Over 12 weeks, both groups completed five weekly sessions incorporating strength, balance, aerobic, and dual-task elements. Significant within-group improvements were observed in the arm curl test (HFG: *p* = 0.031; LFG: *p* = 0.002), chair stand (LFG: *p* < 0.001), and 2 min step test (LFG: *p* = 0.002). Between-group analysis showed greater percentage gains in the LFG for the chair stand (33.8% ± 28.2% vs. 7.1% ± 21.6%, *p* = 0.001) and step test (13.7% ± 14.5% vs. 5.3% ± 14.7%, *p* = 0.040). No significant changes were found in handgrip strength, gait speed, pulse wave velocity, or muscle mass. These results suggest that the modified Vivifrail protocol enhances lower-limb endurance in lower-fitness individuals, but additional adaptations may be necessary to impact vascular and compositional outcomes.

## 1. Introduction

The global aging population has led to a sharp increase in age-related syndromes such as frailty, sarcopenia, and cognitive impairment, which significantly impair independence, mobility, and overall quality of life in older adults. Taiwan, as one of the fastest-aging societies in the world, is projected to become a “super-aged society” in 2025, when more than 20% of its population will be aged 65 and above. According to the National Development Council, Taiwan’s total population is expected to decline from 23.4 million in 2024 to 14.97 million by 2070, underscoring an urgent need for preventive strategies to delay functional decline and enhance health span [1].

Frailty, defined as a state of increased vulnerability to adverse health outcomes, has been associated with higher risks of disability, hospitalization, falls, and mortality [2,3]. Furthermore, the co-occurrence of sarcopenia—a progressive loss of muscle mass and strength—with frailty exacerbates the risk of disability and impairs daily function. Research shows that muscle mass begins to decline at a rate of 8% per decade after age 40, accelerating to 15% per decade after 70 years of age [4].

To address these challenges, the World Health Organization (WHO) recommends that older adults engage in at least 150–300 min of moderate-intensity aerobic activity or 75–150 min of vigorous-intensity activity per week, combined with strength training for major muscle groups on two or more days. These guidelines emphasize balance and strength activities to prevent falls and functional decline.

In response, the Vivifrail program, promoted across Europe and adapted in Taiwan by the Health Promotion Administration, offers a structured and tiered exercise intervention tailored to the physical capacities of older adults [5]. It classifies participants using the Short Physical Performance Battery (SPPB) into six levels (A to D), where D-level represents the healthiest subgroup of older adults with no major mobility impairments. Despite its comprehensive design incorporating resistance, aerobic, balance, and flexibility training, growing evidence suggests that the standard Vivifrail D-level module may be overly conservative for this group. This leads to ceiling effects, especially in those with higher baseline physical function [6]. Prior research involving high-functioning older adults has similarly reported limited gains from standard exercise protocols, underscoring the importance of tailored interventions. For example, studies by Monteiro et al. (2023) and Ferrari et al. (2016) demonstrated that high-capacity older adults often require higher training intensities or novel stimuli (e.g., dual-task or power-based components) to elicit meaningful physiological and functional improvements [7,8]. The 30 s chair stand test is a validated measure of lower-limb muscular endurance and has been shown to correlate strongly with physical independence and fall risk in older adults [9]. From a physiological perspective, individuals with lower baseline muscular capacity often demonstrate a greater adaptive response to resistance and multicomponent training, consistent with the principle of initial values—whereby those with poorer initial function tend to show more pronounced improvements [10,11]. This stratification approach allows us to explore how baseline fitness influences functional gains in response to an upgraded Vivifrail intervention.

Field observations in Taiwan have shown that some robust older adults participating in the D-level Vivifrail program experience limited improvements in mobility, with occasional declines in walking speed. This suggests that the default D-level protocol may lack sufficient intensity or volume to elicit meaningful gains in more robust older adults. Such findings underscore the importance of adjusting exercise prescriptions according to physical capacity, ensuring sufficient overload for adaptation as recommended by the American College of Sports Medicine (ACSM) [12].

The current study aims to evaluate an advanced adaptation of the Vivifrail D-level module, involving increased training frequency (five sessions per week), session duration (120 min per coached session), and exercise diversity (including dual-task elements and dynamic balance training). This study focuses on healthy D-level older adults in Taiwan, stratified into an HFG and LFG based on sit-to-stand strength criteria. Key outcome measures include arterial stiffness indices, functional fitness, and body composition. This research holds practical relevance for public health planning and community-based aging policies, offering data to inform upgrades to national exercise programs for older adults. In this study, we define “functional adaptations” as improvements in physical capacities that directly support daily functional independence—specifically, muscular strength, endurance, balance, and aerobic capacity. While vascular function and body composition are also assessed, they are considered secondary indicators that may reflect broader systemic adaptations to the intervention.

## 2. Materials and Methods

### 2.1. Study Design

**Figure 1:**
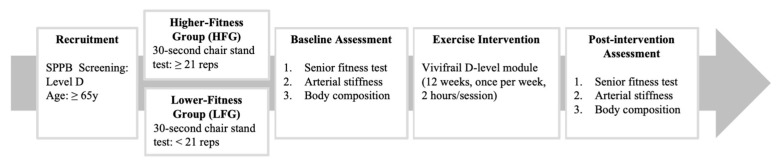
Study design flow chart.

This study adopted a quasi-experimental design with two comparison groups to investigate the effects of an advanced Vivifrail D-level exercise intervention on functional fitness, arterial stiffness, and body composition in community-dwelling older adults. Participants were non-randomly assigned to either the HFG (*n* = 22) or the LFG (*n* = 19) based on their lower-limb muscle strength, stratified by the 30 s chair stand test and SPPB levels (Figure 1). This non-random allocation was based on validated age-specific thresholds from the 30 s chair stand test to stratify participants by baseline lower-limb strength. This design enabled targeted comparisons of physiological responsiveness. Potential selection bias was minimized through strict inclusion/exclusion criteria, standardized measurement conditions, and adjustment for baseline differences via repeated-measures ANOVA.

### 2.2. Participants

Community-dwelling older adults aged ≥65 years (or Indigenous adults ≥55 years) with a D-level classification on the SPPB were recruited from Taipei City. Recruitment was conducted via referrals from three local community health centers and senior activity groups in Taipei City. Participants received verbal and written study explanations before enrollment. Adherence to the home-based sessions was tracked through weekly exercise logs, and supervised session attendance was recorded by research staff. Participants were stratified into two groups based on their performance in the 30 s chair stand test, using age-specific normative thresholds derived from the Senior Fitness Test Manual [13]. Specifically, those performing below 11 repetitions (for ages 65–69), below 10 (ages 70–79), or below 9 (ages 80–84) were categorized into the LFG (*n* = 19), while those meeting or exceeding these thresholds were assigned to the HFG (*n* = 22). All participants were women who were retired, had comparable socio-demographic profiles, and shared similar educational backgrounds—most had completed junior high or high school. They were free from musculoskeletal, neurological, or cardiovascular conditions in the preceding three months and had similar access to community-based health services. This sampling approach helped reduce heterogeneity in lifestyle-related factors that could influence exercise responsiveness. The implications of studying an all-female cohort are further discussed in the Discussion Section. Baseline demographic characteristics were as follows: HFG—71.3 ± 4.3 years, 157.2 ± 6.6 cm, 57.6 ± 6.8 kg, and BMI 23.4 ± 3.6; LFG—73.4 ± 5.4 years, 158.1 ± 7.3 cm, 56.2 ± 7.6 kg, and BMI 20.1 ± 6.4. All participants provided written informed consent. The study protocol was approved by the Research Ethics Committee of National Taiwan University (REC-NTU No. 202312EM073) and conducted in accordance with the Declaration of Helsinki.

### 2.3. Experimental Procedures

All participants completed a 14-week study protocol. The 14-week timeline included baseline assessments in Week 1, a 12-week intervention phase (Weeks 2–13), and post-testing in Week 14. All assessments were conducted at the same time of day (between 9 and 11 AM) and in a fasted but hydrated state, administered by the same team to ensure standardization. Baseline testing was conducted during Week 1, followed by a 12-week intervention (Weeks 2–13) and post-testing in Week 14. The Vivifrail D-level program included five sessions per week: one supervised group session (2 h/week) and four self-directed sessions recorded in exercise logs. The program was designed to progressively target strength, balance, endurance, and flexibility.

### 2.4. Exercise Intervention

The Vivifrail D-level exercise program is a structured multicomponent intervention tailored for robust older adults to enhance functional capacity and prevent frailty. Implemented over 12 weeks, the program comprises five sessions per week: one supervised group session (2 h) and four self-administered home sessions. The supervised sessions follow the Vivifrail D-level circular training structure, incorporating strength, balance, and functional mobility exercises.

The training protocol included the following core components: walking (20 min × 2 sets), sit-to-stand exercises (3 sets of 12 reps), water bottle lifts (3 sets of 12 reps), towel wringing for grip strength (3 sets of 12 reps), upper-body reaching and arm extension (3 sets of 9 reps), forward bending and leg stretches (6 sets of 10 s), stair climbing (3 sets of 20 steps), balloon tapping with walking (3 sets of 10 steps), and figure-8 walking patterns (2 sets of 2 laps).

From Week 1 to Week 6, the focus was on developing technique, rhythm, and joint control. From Week 7 onward, the walking component was progressively increased to 30–45 min, and additional dual-task elements (e.g., memory recall during walking) and dynamic balance tasks (e.g., tandem stance turns) were introduced. This structured weekly progression reflects the principle of progressive overload, wherein motor complexity, training duration, and cognitive demand are gradually increased. Intensity was self-monitored using the “talk test” to ensure safety and maintain moderate exertion throughout the program [6]. This approach aims to improve cardiovascular endurance, muscular strength, flexibility, coordination, and balance in older adults.

### 2.5. Outcome Measures

Outcome domains were selected to reflect primary targets of the Vivifrail program: functional fitness for independence, vascular health for cardiovascular resilience, and muscle mass for sarcopenia prevention. Outcome measures were grouped into four major domains to evaluate the effects of the exercise intervention.

#### 2.5.1. Senior Fitness Test

Physical function was assessed using the Senior Fitness Test [14], which includes seven standardized components: grip strength, bicep curl (upper-limb endurance), 4 m gait speed, 30 s chair stand (lower-limb strength), one-leg stance (static balance), 8-foot up-and-go test (agility), and 2 min step test (cardiorespiratory endurance) (Senior Fitness Test System, Accuratus Corp., Taipei, Taiwan). These tests collectively evaluate key domains of mobility, muscular strength, balance, and coordination relevant to independent living in older adults.

The Senior Fitness Test has been validated in community-dwelling older adults across diverse populations, with intraclass correlation coefficients (ICCs) typically exceeding 0.80 for key components such as the chair stand, arm curl, and up-and-go tests [13,15]. Regionally, normative data have been established in Taiwan [16] and Hong Kong [17], supporting its applicability among older adults of Chinese ethnicity, particularly those residing in East Asian regions. These regional validations reinforce the cross-cultural reliability of the Senior Fitness Test and support its use in evaluating functional fitness in Chinese cultural contexts.

#### 2.5.2. Arterial Stiffness

Peripheral arterial stiffness and blood pressure indices were evaluated using the Omron HBP-8000 (Omron HBP-8000, Omron Healthcare Co., Kyoto, Japan), including brachial–ankle pulse wave velocity (baPWV) and the ankle–brachial index (ABI) bilaterally. The Omron HBP-8000 device has demonstrated good test–retest reliability and validity in older adults for baPWV and ABI measurement [18].

#### 2.5.3. Body Composition

Body composition was evaluated using two devices. Muscle mass, fat mass, and segmental body composition (arms and thighs) were measured via dual-energy X-ray absorptiometry (GE Lunar iDXA, GE Healthcare, Madison, WI, USA) before and after the intervention. The GE Lunar iDXA is considered the gold standard for body composition analysis with excellent inter-device consistency and a low coefficient of variation [19,20].

#### 2.5.4. Short Physical Performance Battery

The SPPB was administered by two trained and quality-assured assessors (ICC = 0.81). It includes three timed components: a 4 m usual gait speed test, a five-repetition chair stand, and a progressive standing balance test [21]. Each component was scored from 0 to 4, with higher scores indicating better performance. The best of two walking trials was recorded. Total scores ranged from 4 to 12 and were categorized—4–6 (low performance), 7–9 (moderate performance), and 10–12 (high performance)—based on clinically validated cut-off points [22].

The SPPB has demonstrated strong test–retest and inter-rater reliability in older adult populations, with ICC values exceeding 0.80 for individual components such as gait speed and chair stand [23]. The test is widely regarded as a robust predictor of disability, hospitalization risk, and all-cause mortality [24], making it a valid and reliable indicator of lower-extremity function in community-based aging research.

#### 2.5.5. Assessment Standardization and Rater Reliability

All outcome assessments were conducted under standardized conditions to minimize potential sources of bias. Testing was performed in the morning hours (between 9:00 and 11:30 AM) to reduce circadian variation. Participants were instructed to avoid strenuous activity the day before testing and were assessed at least two hours after a light breakfast to maintain similar nutritional status. Environmental factors such as room temperature (maintained at 23 ± 1 °C) and noise level were controlled during testing.

All performance-based tests were administered by a team of assessors who completed a standardized training protocol that included detailed instruction, practical demonstrations, and supervised mock assessments. Inter-rater reliability for key outcome measures (e.g., SPPB, chair stand, and gait speed) was evaluated prior to data collection, with intraclass correlation coefficients (ICCs) ≥ 0.80, confirming measurement consistency.

#### 2.5.6. Vivifrail Classification

Based on the total SPPB score, participants were classified into Vivifrail functional levels, with D-level (score 10–12) representing robust or healthy older adults. Only individuals meeting the D-level criteria were included in this study.

### 2.6. Data Availability

The datasets generated and analyzed during the current study are available from the corresponding author upon reasonable request.

### 2.7. Statistical Analysis

All data are expressed as the mean ± standard deviation. Statistical analyses were performed using GraphPad Prism 8.0. The normality of distributions was tested using the Shapiro–Wilk test. The homogeneity of variance and sphericity were assessed via Levene’s and Mauchly’s tests. A two-way repeated-measures ANOVA was used to compare the main effects of time (pre/post) and group (HFG vs. LFG), and Sidak’s post hoc comparisons were conducted where interactions were significant. Results were also normalized as percentage change [(Post − Pre)/Pre × 100%], with statistical significance set at α = 0.05. Prior to conducting the two-way repeated-measures ANOVA, the Shapiro–Wilk test was used to examine normality, Levene’s test assessed the homogeneity of variances, and Mauchly’s test evaluated sphericity. Greenhouse–Geisser corrections were applied when sphericity was violated. Effect sizes (partial eta-squared, η^2^) were reported for significant ANOVA effects, and 95% confidence intervals were included for post hoc comparisons.

## 3. Results

### 3.1. Intervention Effects on Vascular, Functional, and Muscular Health

As shown in Table 1, both groups demonstrated reductions in PWV on the right and left sides following the intervention, although these changes did not reach statistical significance. A significant decrease in the left ABI was observed in the LFG (*p* < 0.05). Regarding functional fitness, the arm curl test significantly improved in both the HFG and LFG (*p* < 0.05), while additional gains were observed in the LFG for the chair stand test and the 2 min step test (*p* < 0.05). No significant changes were found in handgrip strength, 8-foot up-and-go, or 4 m walk tests in either group. Body composition measures, including the skeletal muscle index (SMI) and muscle mass of the upper and lower limbs, showed no significant changes post-intervention. Overall, the Vivifrail D-level program elicited meaningful improvements in muscular endurance but had minimal impact on vascular stiffness and muscle mass within the 12-week intervention period. All participants completed the 12-week intervention. The average supervised session attendance rate was 95.2%, and the completion rate for self-administered home sessions was 89.4%. No participants dropped out of the study, indicating high adherence to and the feasibility of the upgraded Vivifrail D-level protocol. For significant within-group changes, partial eta-squared values ranged from 0.14 to 0.21, indicating moderate effect sizes.

Although the observed changes in PWV and muscle mass did not reach statistical significance, a downward trend in PWV and a slight increase in muscle mass in the LFG may suggest a positive trajectory. These findings, while preliminary, could hold potential clinical relevance in the context of early intervention for vascular health and sarcopenia prevention in robust older adults.

### 3.2. Improvements in Arterial Stiffness Indices

**Figure 2:**
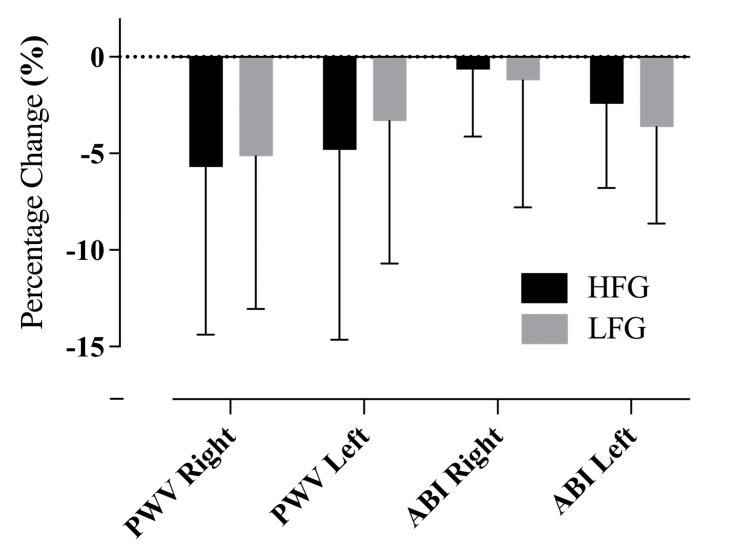
Percentage changes in arterial stiffness indices after the 12-week Vivifrail D-level exercise intervention. Values are presented as mean ± standard deviation. HFG = higher-fitness group; LFG = lower-fitness group; PWV = pulse wave velocity; ABI = ankle–brachial index. No significant between-group differences were observed (*p* > 0.05).

Following the 12-week Vivifrail D-level intervention, a significant within-group reduction in the left ABI was observed in the LFG (from 1.14 ± 0.05 [95% CI 1.117–1.163] to 1.10 ± 0.06 [95% CI 1.073–1.127], *p* = 0.0050, η^2^ = 0.3622), indicating a large effect size. Meanwhile, no significant changes were noted in PWV or the right ABI within either group (Table 1). When expressed as a percentage change from baseline, there were no significant between-group differences in right PWV (HFG: −5.7% ± 8.7% vs. LFG: −5.1% ± 7.9%, *p* = 0.416), left PWV (HFG: −4.8% ± 9.8% vs. LFG: −3.3% ± 7.4%, *p* = 0.296), the right ABI (HFG: −0.6% ± 3.5% vs. LFG: −1.2% ± 6.6%, *p* = 0.374), or the left ABI (HFG: −2.4% ± 4.4% vs. LFG: −3.6% ± 5.0%, *p* = 0.218) (Figure 2).

Although these changes were not statistically significant, the consistent trend toward decreased PWV values in both groups may indicate early-stage vascular adaptations, particularly in a preventive health framework.

### 3.3. Functional Fitness Gains Across Groups

Following the 12-week Vivifrail D-level intervention, both the HFG and LFG exhibited significant within-group improvements in selected functional fitness measures. The HFG showed a significant improvement in the arm curl test (21.2 ± 3.7 [95% CI 19.61–22.85] to 23.5 ± 4.3 [95% CI 21.58–25.42], *p* = 0.0119, η^2^ = 0.2655), indicating a moderate-to-large effect size. In the LFG, significant enhancements were observed in the arm curl test (17.5 ± 3.8 [95% CI 15.64–19.31] to 21.6 ± 4.5 [95% CI 19.40–23.75], *p* = 0.0002, η^2^ = 0.5427), the 30 s chair stand (14.6 ± 2.2 [95% CI 13.56–15.70] to 19.4 ± 4.7 [95% CI 17.11–21.62], *p* < 0.0001, η^2^ = 0.6187), and the 2 min step test (92.3 ± 10.6 [95% CI 87.22–97.41] to 102.5 ± 11.7 [95% CI 96.82–108.13], *p* = 0.0009, η^2^ = 0.4647), all indicating large effect sizes (Table 1).

**Figure 3:**
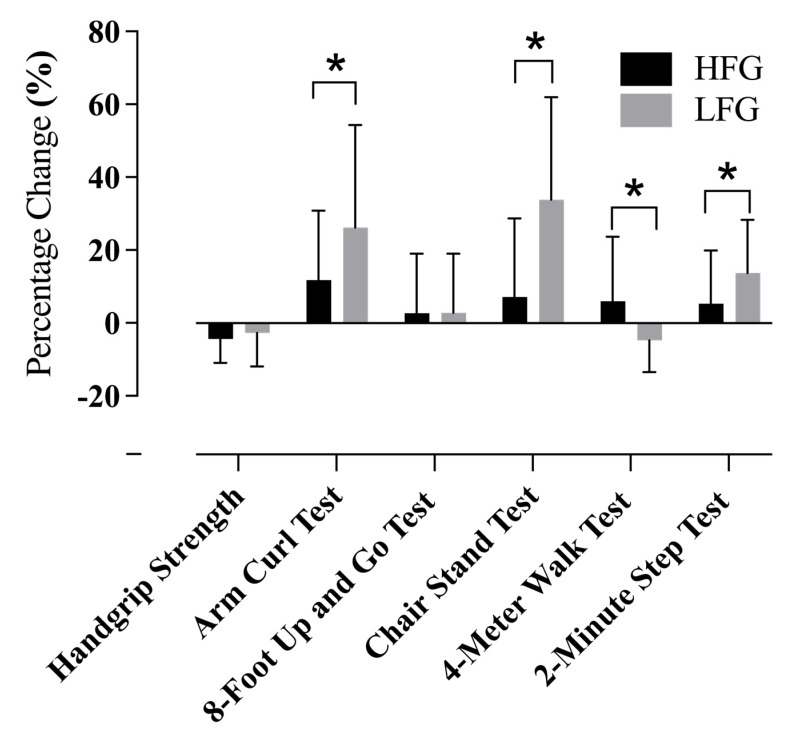
Percentage changes in functional fitness measures following the 12-week Vivifrail D-level exercise intervention. Data are presented as mean ± standard deviation. HFG = higher-fitness group; LFG = lower-fitness group. * indicates a significant difference between HFG and LFG (*p* < 0.05).

Between-group comparisons of percentage improvements revealed that the LFG demonstrated significantly greater gains in arm curl performance (11.8 ± 19.1 [95% CI 3.28–20.22] to 26.2 ± 28.1 [95% CI 12.64–39.75], *p* = 0.0294, η^2^ = 0.0885) and 30 s chair stand performance (7.1 ± 21.6 [95% CI −2.42–16.71] to 33.8 ± 28.2 [95% CI 20.19–47.39], *p* = 0.0007, η^2^ = 0.2310), indicating small-to-moderate and moderate effect sizes, respectively. Conversely, the HFG demonstrated significantly greater improvement in the 4 m walk test compared to the LFG (from −4.7% ± 8.7 [95% CI −1.34–14.73] to 5.9% ± 17.7 [95% CI −8.43–0.93], *p* = 0.0152, η^2^ = 0.1271), reflecting a small-to-moderate effect size. Additionally, the LFG showed a significantly greater percentage improvement in the 2 min step test (5.3 ± 14.7 [95% CI −1.39–11.96] to 13.7 ± 14.5 [95% CI 6.51–20.91], *p* = 0.0398, η^2^ = 0.0807), also indicating a small-to-moderate effect size. No significant between-group differences were observed in grip strength (*p* = 0.279) or the 8-foot up-and-go test (*p* = 0.492) (Figure 3).

### 3.4. Changes in Body Composition Outcomes

**Figure 4:**
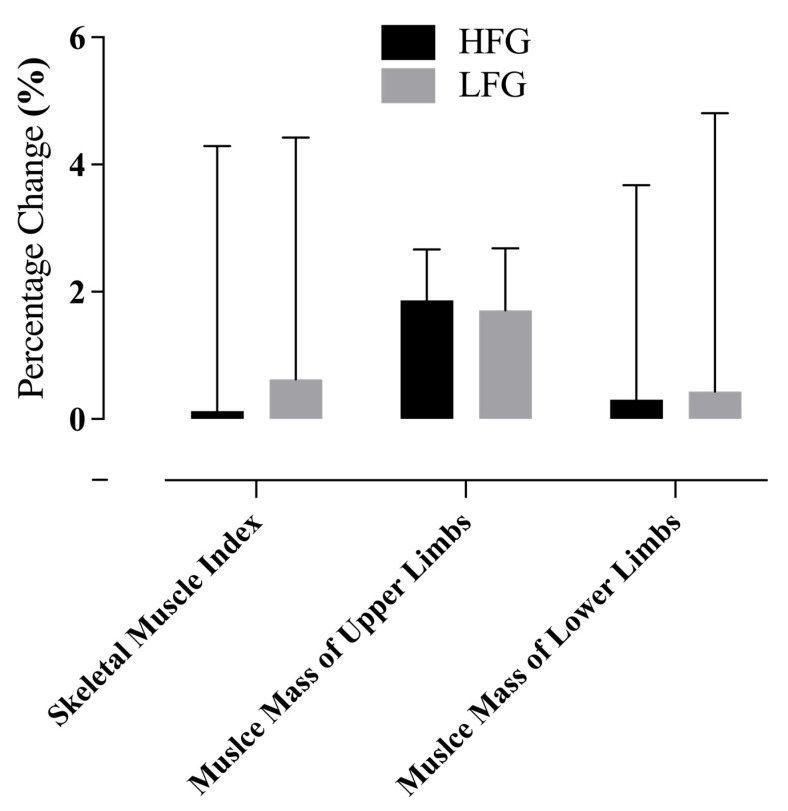
Percentage changes in body composition measures following the 12-week Vivifrail D-level exercise intervention. Data are presented as mean ± standard deviation. HFG = higher-fitness group; LFG = lower-fitness group. No significant between-group differences were observed (*p* > 0.05).

Following the 12-week Vivifrail D-level intervention, no significant within-group or between-group differences were observed in the SMI, muscle mass of upper limbs, or muscle mass of lower limbs. When expressed as a percentage change from baseline, no significant differences were found between the HFG and LFG in the SMI (HFG: 0.1% ± 4.2% [95% CI −0.89–1.16] vs. LFG: 0.6% ± 3.8% [95% CI −0.15–1.01], *p* = 0.360, η^2^ = 0.0233), muscle mass of upper limbs (HFG: −1.6% ± 5.1% [95% CI −4.25–0.65] vs. LFG: 0.3% ± 3.4% [95% CI −1.98–1.46], *p* = 0.183, η^2^ = 0.0248), or muscle mass of lower limbs (HFG: −0.3% ± 3.1% [95% CI −2.31–1.31] vs. LFG: 0.4% ± 4.4% [95% CI −2.16–2.10], *p* = 0.461, η^2^ = 0.0003) (Figure 4).

Notably, while the increase in the SMI and limb muscle mass in the LFG did not reach significance, the direction of change suggests a possible attenuation of age-related muscle loss. Such subtle adaptations, if sustained over longer periods, may be clinically meaningful in mitigating sarcopenia-related risks.

## 4. Discussion

This study investigated the effects of an advanced Vivifrail D-level exercise intervention on arterial stiffness, functional fitness, and body composition in robust older adults with different baseline fitness levels. The findings showed selective functional gains, particularly in muscular endurance and lower-limb strength, but limited changes in vascular and compositional outcomes.

A significant within-group improvement was observed in the left ABI among participants in the LFG. However, PWV and right ABI values did not significantly change in either group. These findings are consistent with previous evidence indicating that short-term multicomponent exercise programs exert minimal effects on arterial stiffness in healthy older adults, especially those who already exhibit optimal vascular function [7,25]. Previous studies have also suggested that longer-duration or higher-intensity aerobic interventions may be required to elicit measurable vascular adaptations in this population [26]. Nonetheless, it is worth noting that both the HFG and LFG exhibited a downward trend in pulse wave velocity values, which, although not statistically significant, may indicate early vascular responsiveness to multicomponent exercise. In a clinical context, such modest shifts—particularly in robust older adults with already normative ABI and PWV values—could reflect preclinical adaptations that warrant further exploration in longer interventions.

Substantial improvements in functional fitness were observed following the intervention. Both HFG and LFG participants showed significant gains in the arm curl test, with the LFG also exhibiting notable improvements in the 30 s chair stand and 2 min step tests. Furthermore, between-group comparisons demonstrated significantly greater percentage changes in multiple functional indicators among the LFG, emphasizing that older adults with lower baseline physical capacity tend to experience more marked benefits from exercise interventions [27]. These results reinforce the principle that functional adaptations are more pronounced when initial fitness is lower, as supported by previous multicomponent training research [28]. This may be due to greater physiological reserve and adaptive potential in individuals with lower baseline fitness, where neuromuscular systems are more responsive to training stimuli. The principle of initial value and the concept of ceiling effects in high-functioning older adults support this observation.

Interestingly, no significant improvements were noted in mobility-related outcomes, including the 8-foot up-and-go and 4 m walk tests, despite observed gains in muscular strength and endurance. This finding highlights a critical gap between improvements in isolated physical fitness domains and their translation to complex motor tasks that require neuromuscular coordination and executive control. Previous research suggests that mobility in older adults is influenced not only by physical strength but also by cognitive capacity and motor planning ability, elements not directly targeted by traditional exercise programs [29].

To address these limitations, incorporating dual-task training—which combines physical and cognitive demands—has emerged as an effective strategy to enhance balance and mobility. For example, Eggenberger et al. demonstrated that a 16-week dual-task training protocol significantly improved gait speed and reduced fall risk in older adults [30]. Similarly, a meta-analysis by Silsupadol et al. supported the effectiveness of cognitive–motor dual-task exercises in improving dynamic balance among older populations [31]. Future Vivifrail protocols should consider integrating dual-task components to address the multidimensional nature of functional mobility in aging.

In terms of body composition, no significant changes were observed in the SMI, arm muscle mass, or thigh muscle mass in either group. However, a small positive change in the SMI and lower-limb muscle mass was noted in the LFG, suggesting a possible attenuation of age-related muscle loss. Although not statistically significant, this trend may be clinically meaningful in maintaining physical function and reducing sarcopenia risk in aging populations. This finding is consistent with the previous literature indicating that moderate-intensity multicomponent training over a 12-week period may be insufficient to stimulate significant hypertrophic responses in older adults [8]. To promote muscle mass gains in older adults, longer or higher-intensity resistance training may be required, as moderate multicomponent programs often fail to induce hypertrophy within 12 weeks. Recent evidence suggests that combining resistance exercise with cognitive tasks can enhance both physical and cognitive outcomes. Baek et al. found that dual-task resistance training led to greater improvements in strength and executive function than traditional protocols in frail older adults [32], highlighting its potential to simultaneously stimulate musculoskeletal and neurological adaptations.

Although a post hoc power analysis indicated that the study was sufficiently powered to detect medium-sized effects (power = 0.80, α = 0.05, f = 0.25) with the available sample (*n* = 41), several limitations should be acknowledged. These include the quasi-experimental design, the relatively small sample size, and the self-selection bias that may have occurred due to self-administered home training sessions. Such factors may limit the generalizability of the findings and the ability to detect more subtle differences. Additionally, as the study included only healthy older women, the findings may not be generalizable to older men or mixed-gender populations. Future research should aim to include more diverse samples to enhance representativeness. Moreover, given the variability in functional outcomes among older adults, larger-scale trials are warranted to validate the observed trends, detect smaller effect sizes, and strengthen the external validity of these results.

In summary, although the advanced Vivifrail D-level program yielded significant improvements in muscular endurance and strength, its limited effects on mobility, vascular function, and body composition highlight the importance of considering clinically meaningful trends beyond statistical significance. Improvements in functional ability, particularly in strength and endurance, are closely linked to daily autonomy and reduced fall risk. Even modest gains can help preserve independence and improve quality of life in older adults, making these trends clinically meaningful despite statistical limitations. These findings underscore the potential value of early functional gains and subtle physiological shifts, which may serve as foundations for more pronounced adaptations with extended or intensified protocols. Combining dual-task approaches and resistance-based hypertrophy strategies may enhance the overall effectiveness of community-based training programs for healthy older adults.

## 5. Conclusions

This study demonstrates that an intensified Vivifrail D-level exercise intervention can effectively improve upper- and lower-limb muscular endurance and functional fitness in healthy older adults, particularly among those with lower baseline strength levels. While meaningful gains in functional outcomes such as the arm curl, chair stand, and step tests were observed, the intervention elicited limited effects on arterial stiffness and body composition over the 12-week period. The lack of improvements in mobility-related tasks and muscle mass highlights the need to further tailor exercise protocols based on participants’ initial functional status. Future adaptations of the Vivifrail program should consider integrating dual-task training and progressive resistance modalities to enhance neuromuscular complexity, promote hypertrophic adaptation, and optimize functional mobility in aging populations. These findings contribute valuable evidence to the design of community-based programs that aim to maintain physical independence and extend health span in an increasingly aging society.

## Figures and Tables

**Table 1:** Effects of the Vivifrail D-level program on vascular, functional, and muscular health indicators.

	HFG—Pre (*n* = 22)	HFG—Post (*n* = 22)	LFG—Pre (*n* = 19)	LFG—Post (*n* = 19)
**Arterial Stiffness**				
Pulse Wave Velocity—Right	1824.8 ± 414.5	1709.5 ± 343.5	1731.8 ± 281	1651.2 ± 265.2
Pulse Wave Velocity—Left	1817.3 ± 360.8	1681.2 ± 323.6	1716.8 ± 309.4	1649.4 ± 264.1
Ankle–Brachial Index—Right	1.14 ± 0.06	1.16 ± 0.08	1.12 ± 0.07	1.11 ± 0.07
Ankle–Brachial Index—Left	1.16 ± 0.08	1.14 ± 0.07	1.14 ± 0.05	1.10 ± 0.06 *
**Functional Fitness**				
Handgrip Strength (kg)	25.7 ± 6.3	23.9 ± 6.1	23.1 ± 7.6	22.2 ± 6.4
Arm Curl Test (repetitions)	21.2 ± 3.7	23.3 ± 4.1 *	17.5 ± 3.8	21.6 ± 4.5 *
8-Foot Up-and-Go Test (seconds)	5.7 ± 1.1	5.7 ± 0.8	6.2 ± 1.2	6.3 ± 1.1
Chair Stand Test (repetitions)	21.7 ± 3.4	22.8 ± 4.7	14.6 ± 2.2	19.5 ± 4.6 *
4-Meter Walk Test (seconds)	2.7 ± 0.5	2.9 ± 0.7	3.0 ± 0.4	2.9 ± 0.6
2-Minute Step Test (steps)	101.8 ± 16.2	105 ± 13.2	92.3 ± 10.6	103 ± 11.3 *
**Body Composition**				
Skeletal Muscle Index	6.3 ± 0.7	6.3 ± 0.6	5.9 ± 0.6	6.1 ± 0.6
Muscle Mass of Upper Limbs (g)	3534.9 ± 677.0	3477.7 ± 584.1	3352.6 ± 565.2	3395.2 ± 553.2
Muscle Mass of Lower Limbs (g)	11,966 ± 1519.2	11,951.7 ± 1536.7	11,515.6 ± 1541.0	11,721.9 ± 1511.4

The data are represented as the mean ± standard deviation. HFG = higher-fitness group; LFG = lower-fitness group. * indicates a statistically significant within-group difference between pre- and post-test values (α = 0.05).

## Data Availability

The data presented in this study are available on request from the corresponding author. The data are not publicly available due to privacy.
